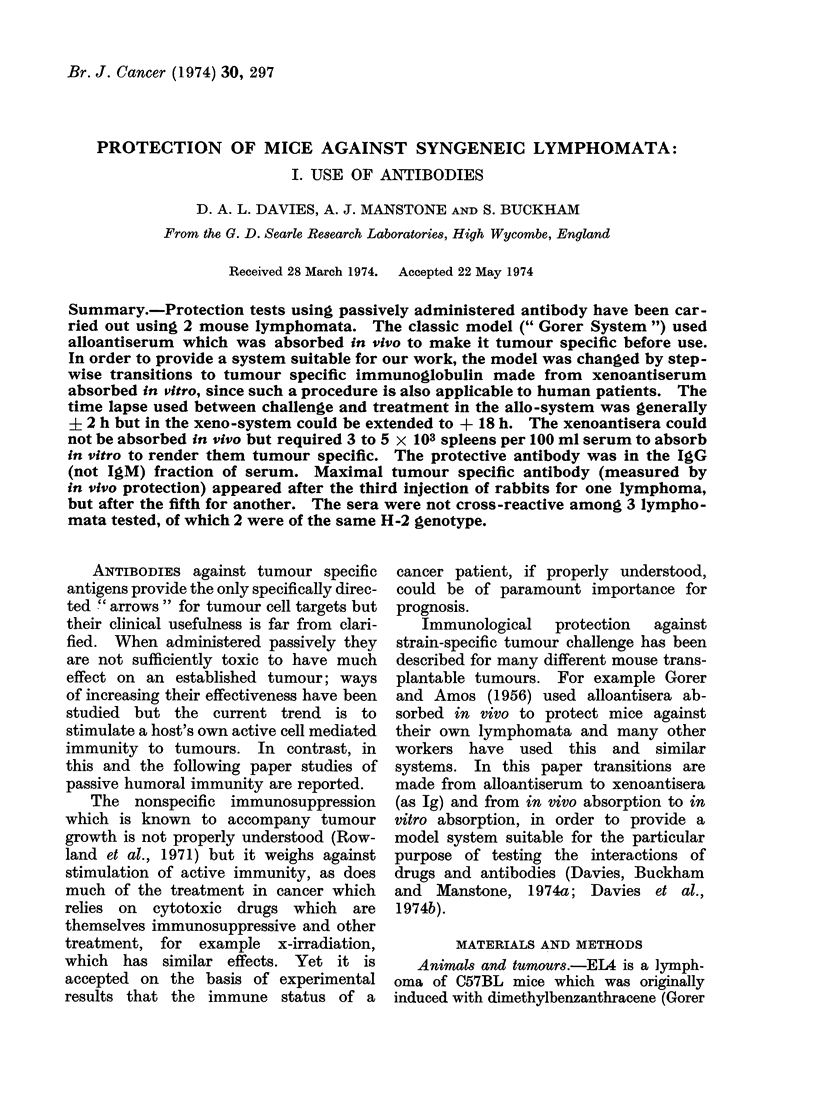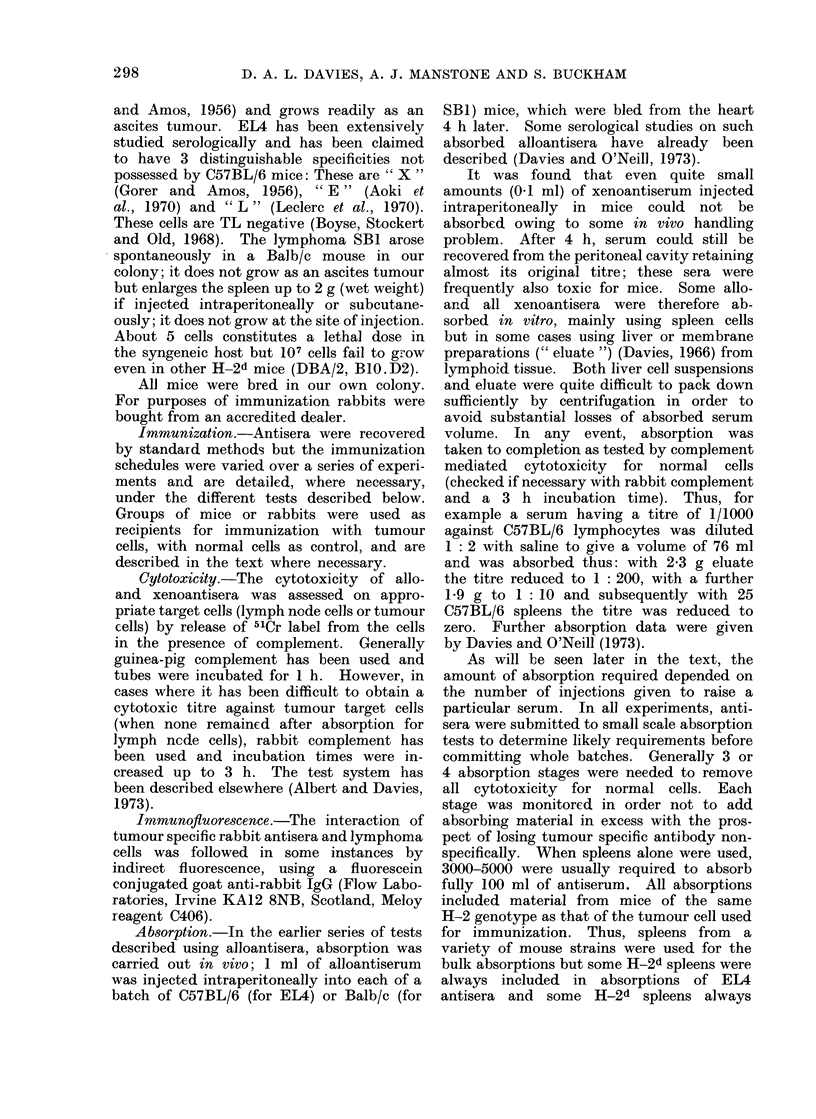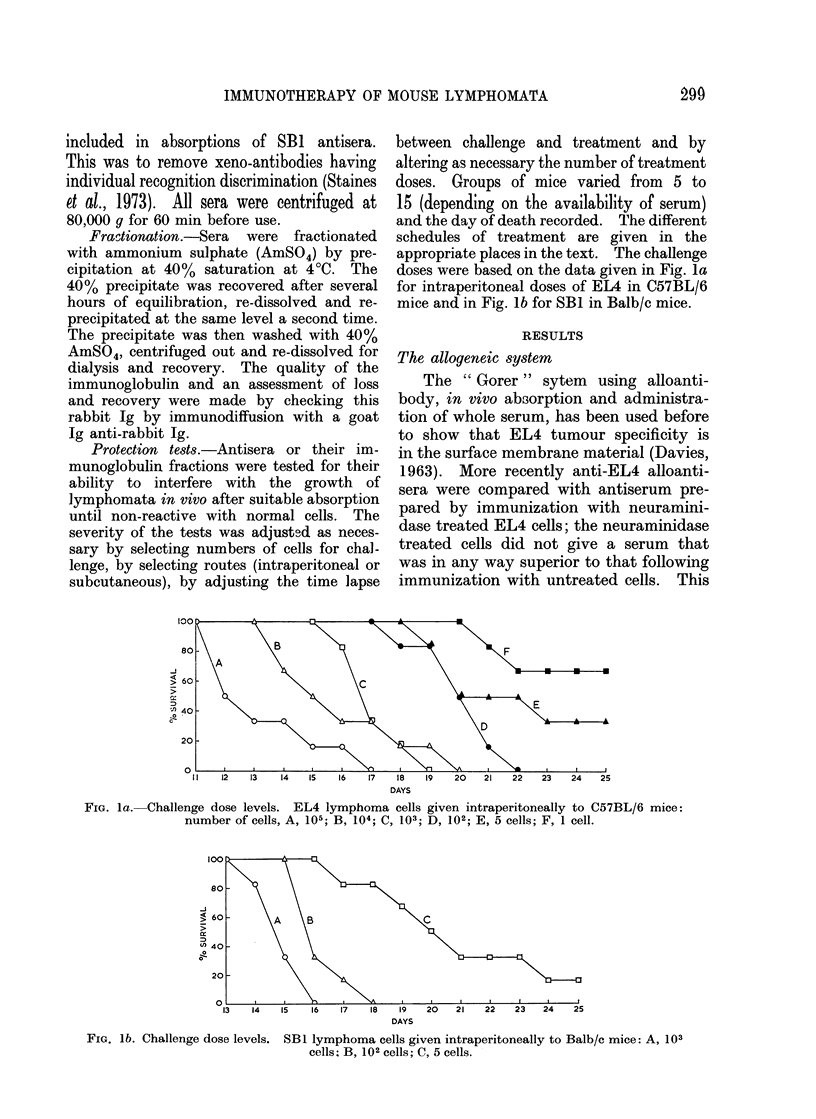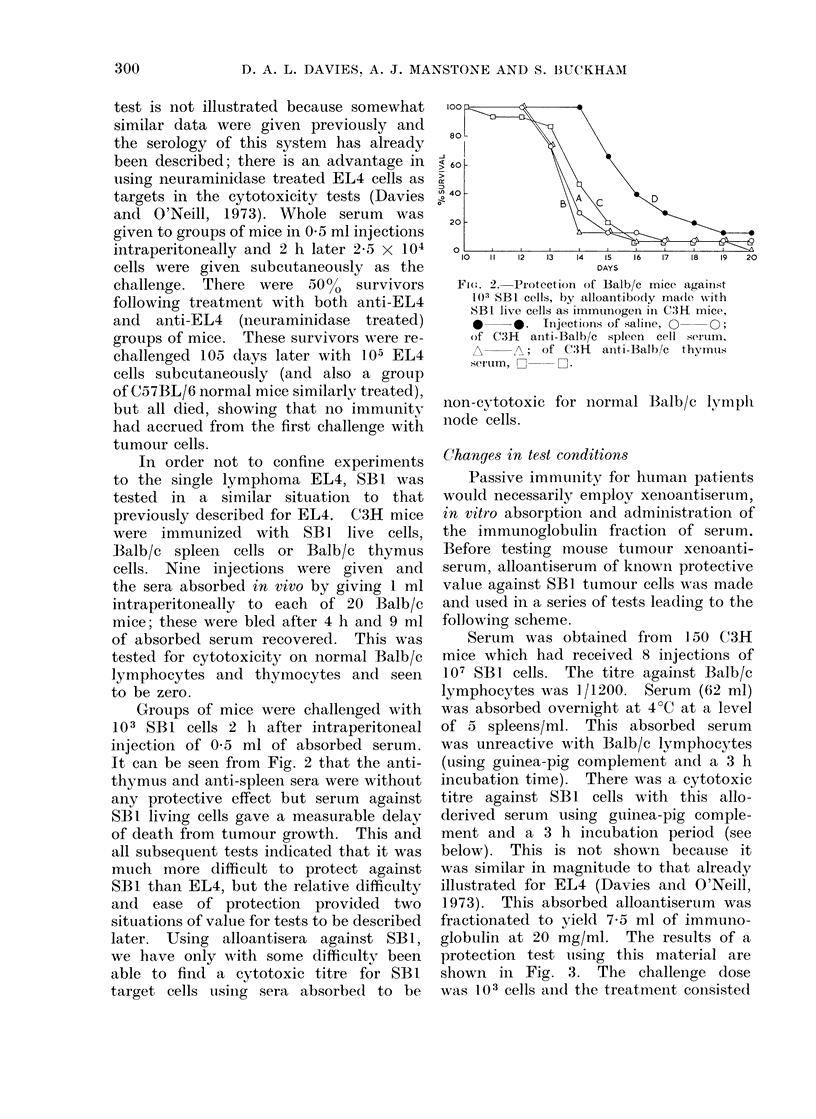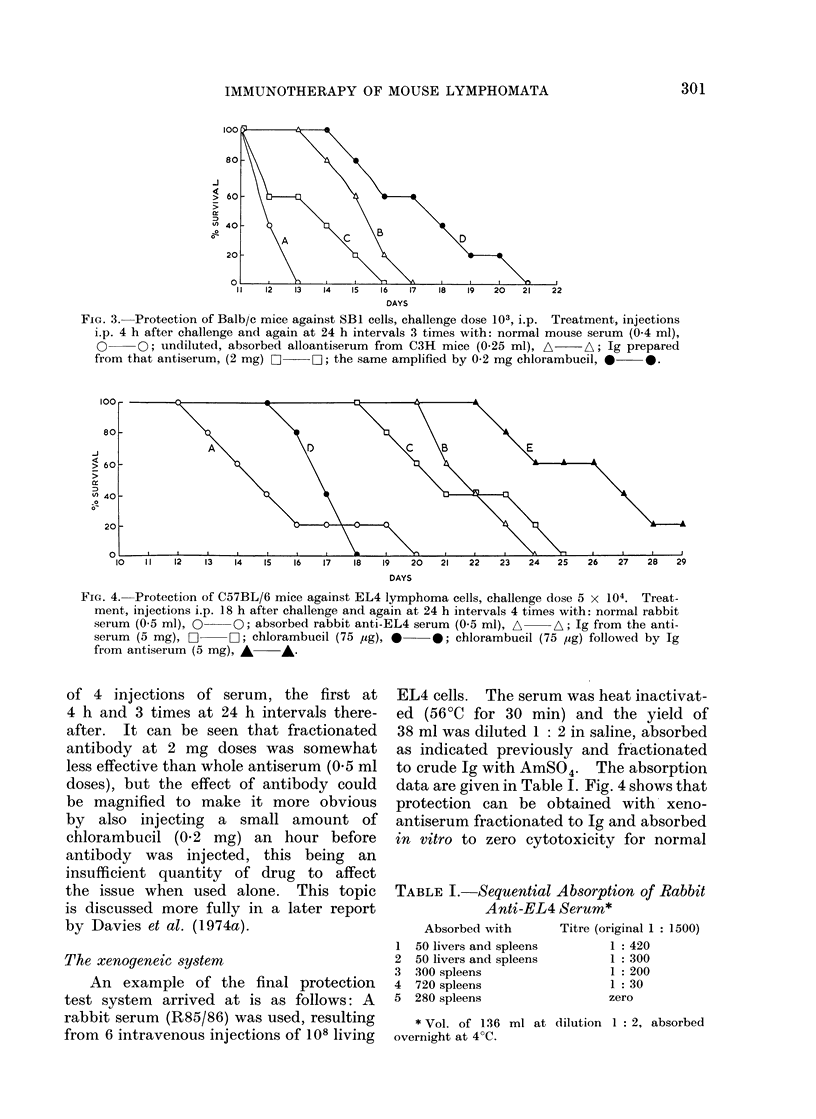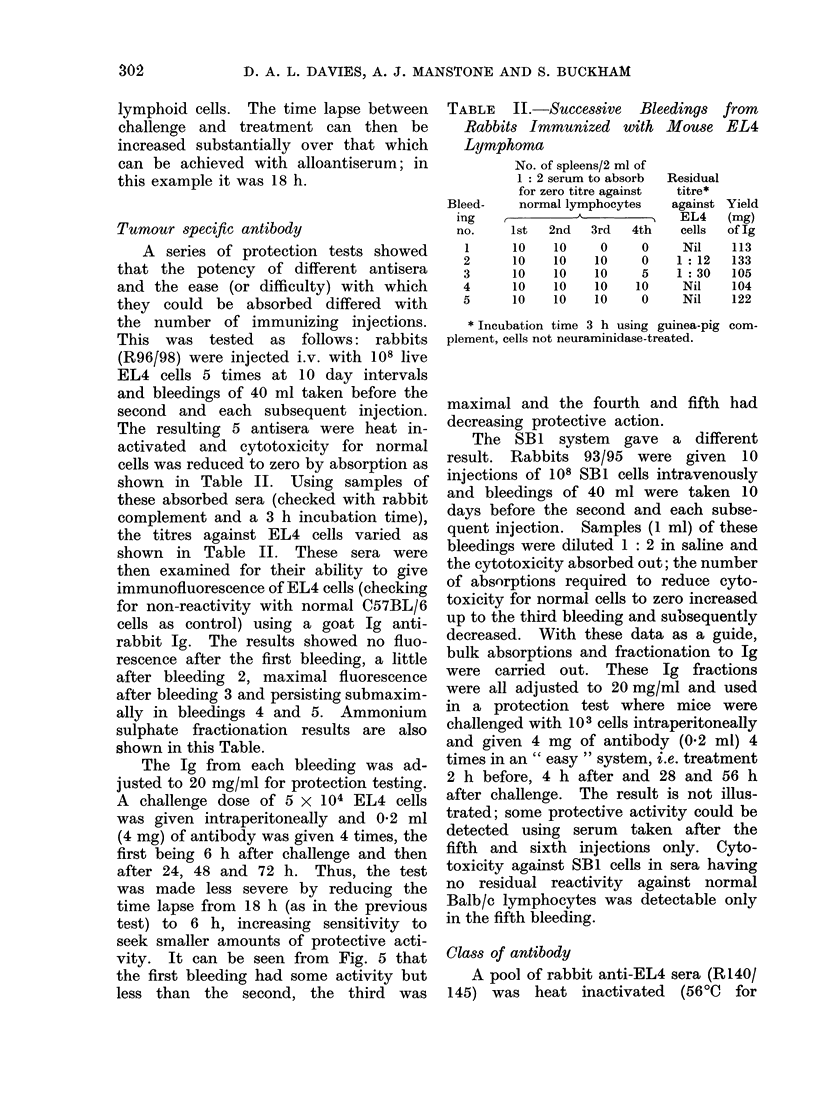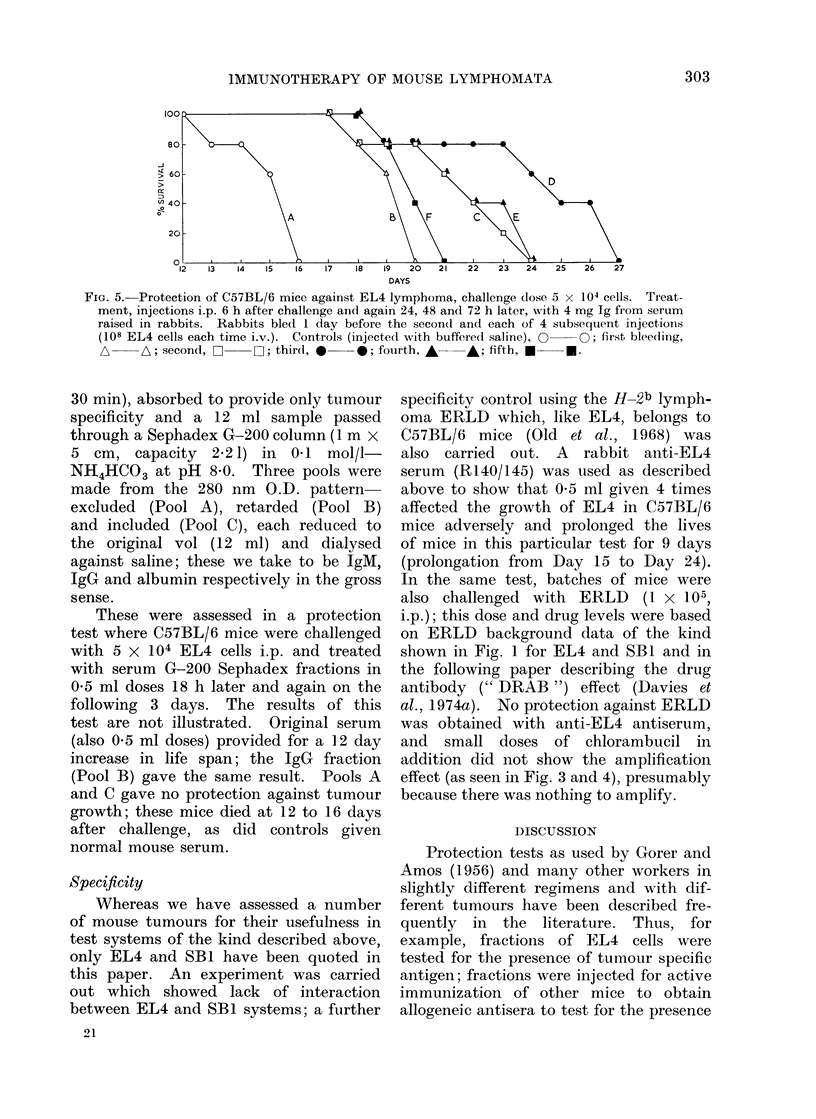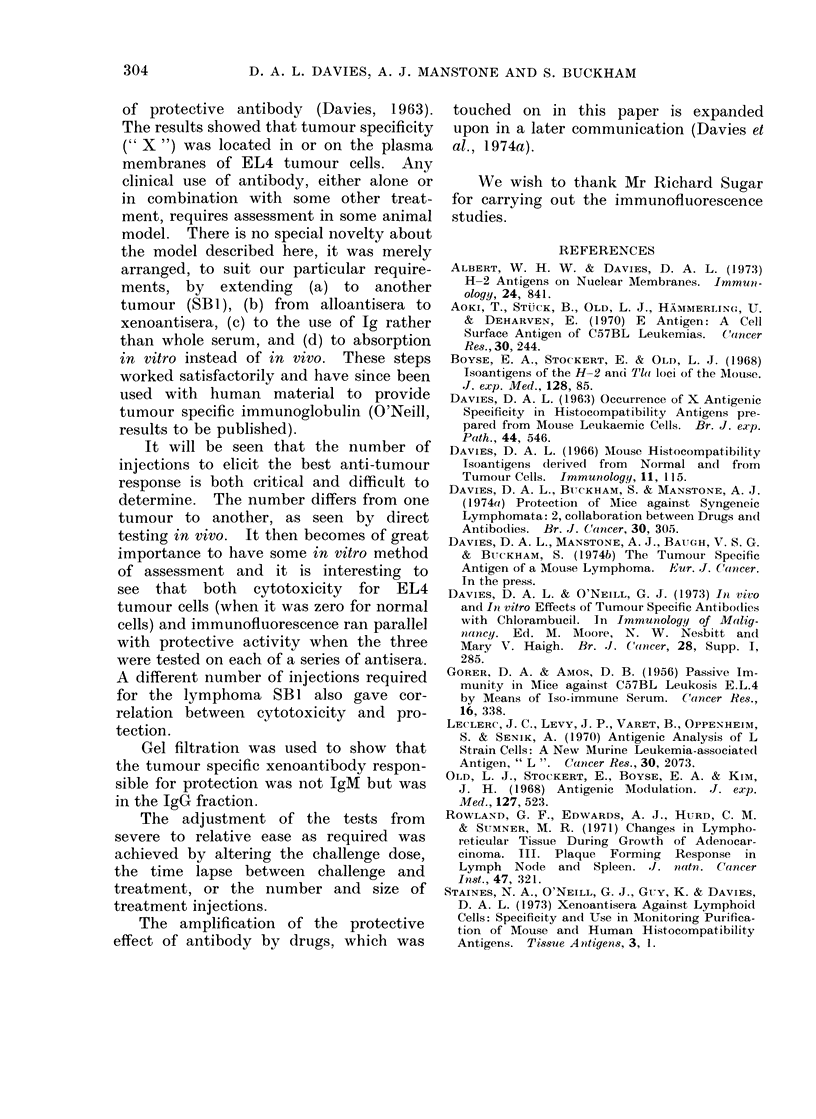# Protection of Mice Against Syngeneic Lymphomata

**DOI:** 10.1038/bjc.1974.196

**Published:** 1974-10

**Authors:** D. A. L. Davies, A. J. Manstone, S. Buckham

## Abstract

Protection tests using passively administered antibody have been carried out using 2 mouse lymphomata. The classic model (“Gorer System”) used alloantiserum which was absorbed *in vivo* to make it tumour specific before use. In order to provide a system suitable for our work, the model was changed by stepwise transitions to tumour specific immunoglobulin made from xenoantiserum absorbed *in vitro,* since such a procedure is also applicable to human patients. The time lapse used between challenge and treatment in the allo-system was generally ± 2 h but in the xeno-system could be extended to + 18 h. The xenoantisera could not be absorbed *in vivo* but required 3 to 5 × 10^3^ spleens per 100 ml serum to absorb *in vitro* to render them tumour specific. The protective antibody was in the IgG (not IgM) fraction of serum. Maximal tumour specific antibody (measured by *in vivo* protection) appeared after the third injection of rabbits for one lymphoma, but after the fifth for another. The sera were not cross-reactive among 3 lymphomata tested, of which 2 were of the same H-2 genotype.


*Br. J. Cancer* (1974) **30**, 297

### PROTECTION OF MICE AGAINST SYNGENEIC LYMPHOMATA: I. USE OF ANTIBODIES

D. A. L. DAVIES, A. J. MANSTONE AND S. BUCKHAM

*From the G. D. Searle Research Laboratories, High Wycombe, England*

Received 28 March 1974. Accepted 22 May 1974

**Summary.—Protection tests using passively administered antibody have been carried out using 2 mouse lymphomata. The classic model (" Gorer System ") used alloantiserum which was absorbed *in vivo* to make it tumour specific before use. In order to provide a system suitable for our work, the model was changed by stepwise transitions to tumour specific immunoglobulin made from xenoantiserum absorbed *in vitro*, since such a procedure is also applicable to human patients. The time lapse used between challenge and treatment in the allo-system was generally $\pm$ 2 h but in the xeno-system could be extended to + 18 h. The xenoantisera could not be absorbed *in vivo* but required 3 to 5 $\times$ $10^3$ spleens per 100 ml serum to absorb *in vitro* to render them tumour specific. The protective antibody was in the IgG (not IgM) fraction of serum. Maximal tumour specific antibody (measured by *in vivo* protection) appeared after the third injection of rabbits for one lymphoma, but after the fifth for another. The sera were not cross-reactive among 3 lymphomata tested, of which 2 were of the same H-2 genotype.**

ANTIBODIES against tumour specific antigens provide the only specifically directed " arrows " for tumour cell targets but their clinical usefulness is far from clarified. When administered passively they are not sufficiently toxic to have much effect on an established tumour; ways of increasing their effectiveness have been studied but the current trend is to stimulate a host's own active cell mediated immunity to tumours. In contrast, in this and the following paper studies of passive humoral immunity are reported.

The nonspecific immunosuppression which is known to accompany tumour growth is not properly understood (Rowland *et al.*, 1971) but it weighs against stimulation of active immunity, as does much of the treatment in cancer which relies on cytotoxic drugs which are themselves immunosuppressive and other treatment, for example x-irradiation, which has similar effects. Yet it is accepted on the basis of experimental results that the immune status of a cancer patient, if properly understood, could be of paramount importance for prognosis.

Immunological protection against strain-specific tumour challenge has been described for many different mouse transplantable tumours. For example Gorer and Amos (1956) used alloantisera absorbed *in vivo* to protect mice against their own lymphomata and many other workers have used this and similar systems. In this paper transitions are made from alloantiserum to xenoantisera (as Ig) and from *in vivo* absorption to *in vitro* absorption, in order to provide a model system suitable for the particular purpose of testing the interactions of drugs and antibodies (Davies, Buckham and Manstone, 1974*a*; Davies *et al.*, 1974*b*).

### MATERIALS AND METHODS

*Animals and tumours.*—EL4 is a lymphoma of C57BL mice which was originally induced with dimethylbenzanthracene (Gorer and Amos, 1956) and grows readily as an ascites tumour. EL4 has been extensively studied serologically and has been claimed to have 3 distinguishable specificities not possessed by C57BL/6 mice: These are " X " (Gorer and Amos, 1956), " E " (Aoki *et al.*, 1970) and " L " (Leclerc *et al.*, 1970). These cells are TL negative (Boyse, Stockert and Old, 1968). The lymphoma SB1 arose spontaneously in a Balb/c mouse in our colony; it does not grow as an ascites tumour but enlarges the spleen up to 2 g (wet weight) if injected intraperitoneally or subcutaneously; it does not grow at the site of injection. About 5 cells constitutes a lethal dose in the syngeneic host but $10^7$ cells fail to grow even in other H–$2^d$ mice (DBA/2, B10.D2).

All mice were bred in our own colony. For purposes of immunization rabbits were bought from an accredited dealer.

*Immunization.*—Antisera were recovered by standard methods but the immunization schedules were varied over a series of experiments and are detailed, where necessary, under the different tests described below. Groups of mice or rabbits were used as recipients for immunization with tumour cells, with normal cells as control, and are described in the text where necessary.

*Cytotoxicity.*—The cytotoxicity of allo- and xenoantisera was assessed on appropriate target cells (lymph node cells or tumour cells) by release of $^{51}Cr$ label from the cells in the presence of complement. Generally guinea-pig complement has been used and tubes were incubated for 1 h. However, in cases where it has been difficult to obtain a cytotoxic titre against tumour target cells (when none remained after absorption for lymph node cells), rabbit complement has been used and incubation times were increased up to 3 h. The test system has been described elsewhere (Albert and Davies, 1973).

*Immunofluorescence.*—The interaction of tumour specific rabbit antisera and lymphoma cells was followed in some instances by indirect fluorescence, using a fluorescein conjugated goat anti-rabbit IgG (Flow Laboratories, Irvine KA12 8NB, Scotland, Meloy reagent C406).

*Absorption.*—In the earlier series of tests described using alloantisera, absorption was carried out *in vivo*; 1 ml of alloantiserum was injected intraperitoneally into each of a batch of C57BL/6 (for EL4) or Balb/c (for SB1) mice, which were bled from the heart 4 h later. Some serological studies on such absorbed alloantisera have already been described (Davies and O'Neill, 1973).

It was found that even quite small amounts (0·1 ml) of xenoantiserum injected intraperitoneally in mice could not be absorbed owing to some *in vivo* handling problem. After 4 h, serum could still be recovered from the peritoneal cavity retaining almost its original titre; these sera were frequently also toxic for mice. Some allo- and all xenoantisera were therefore absorbed *in vitro*, mainly using spleen cells but in some cases using liver or membrane preparations (" eluate ") (Davies, 1966) from lymphoid tissue. Both liver cell suspensions and eluate were quite difficult to pack down sufficiently by centrifugation in order to avoid substantial losses of absorbed serum volume. In any event, absorption was taken to completion as tested by complement mediated cytotoxicity for normal cells (checked if necessary with rabbit complement and a 3 h incubation time). Thus, for example a serum having a titre of 1/1000 against C57BL/6 lymphocytes was diluted 1 : 2 with saline to give a volume of 76 ml and was absorbed thus: with 2·3 g eluate the titre reduced to 1 : 200, with a further 1·9 g to 1 : 10 and subsequently with 25 C57BL/6 spleens the titre was reduced to zero. Further absorption data were given by Davies and O'Neill (1973).

As will be seen later in the text, the amount of absorption required depended on the number of injections given to raise a particular serum. In all experiments, antisera were submitted to small scale absorption tests to determine likely requirements before committing whole batches. Generally 3 or 4 absorption stages were needed to remove all cytotoxicity for normal cells. Each stage was monitored in order not to add absorbing material in excess with the prospect of losing tumour specific antibody non-specifically. When spleens alone were used, 3000–5000 were usually required to absorb fully 100 ml of antiserum. All absorptions included material from mice of the same H–2 genotype as that of the tumour cell used for immunization. Thus, spleens from a variety of mouse strains were used for the bulk absorptions but some H–$2^d$ spleens were always included in absorptions of EL4 antisera and some H–$2^d$ spleens always included in absorptions of SBl antisera. This was to remove xeno-antibodies having individual recognition discrimination (Staines *et al.*, 1973). All sera were centrifuged at 80,000 *g* for 60 min before use.

*Fractionation.*—Sera were fractionated with ammonium sulphate ($AmSO_4$) by precipitation at 40% saturation at 4°C. The 40% precipitate was recovered after several hours of equilibration, re-dissolved and re-precipitated at the same level a second time. The precipitate was then washed with 40% $AmSO_4$, centrifuged out and re-dissolved for dialysis and recovery. The quality of the immunoglobulin and an assessment of loss and recovery were made by checking this rabbit Ig by immunodiffusion with a goat Ig anti-rabbit Ig.

*Protection tests.*—Antisera or their immunoglobulin fractions were tested for their ability to interfere with the growth of lymphomata *in vivo* after suitable absorption until non-reactive with normal cells. The severity of the tests was adjusted as necessary by selecting numbers of cells for challenge, by selecting routes (intraperitoneal or subcutaneous), by adjusting the time lapse between challenge and treatment and by altering as necessary the number of treatment doses. Groups of mice varied from 5 to 15 (depending on the availability of serum) and the day of death recorded. The different schedules of treatment are given in the appropriate places in the text. The challenge doses were based on the data given in Fig. 1*a* for intraperitoneal doses of EL4 in C57BL/6 mice and in Fig. 1*b* for SB1 in Balb/c mice.

### RESULTS

### *The allogeneic system*

The " Gorer " sytem using alloantibody, *in vivo* absorption and administration of whole serum, has been used before to show that EL4 tumour specificity is in the surface membrane material (Davies, 1963). More recently anti-EL4 alloantisera were compared with antiserum prepared by immunization with neuraminidase treated EL4 cells; the neuraminidase treated cells did not give a serum that was in any way superior to that following immunization with untreated cells. This

FIG. 1*a*.—Challenge dose levels. EL4 lymphoma cells given intraperitoneally to C57BL/6 mice: number of cells, A, $10^5$; B, $10^4$; C, $10^3$; D, $10^2$; E, 5 cells; F, 1 cell.

FIG. 1*b*. Challenge dose levels. SB1 lymphoma cells given intraperitoneally to Balb/c mice: A, $10^3$ cells: B, $10^2$ cells; C, 5 cells.

test is not illustrated because somewhat similar data were given previously and the serology of this system has already been described; there is an advantage in using neuraminidase treated EL4 cells as targets in the cytotoxicity tests (Davies and O'Neill, 1973). Whole serum was given to groups of mice in 0·5 ml injections intraperitoneally and 2 h later $2{\cdot}5 \times 10^4$ cells were given subcutaneously as the challenge. There were 50% survivors following treatment with both anti-EL4 and anti-EL4 (neuraminidase treated) groups of mice. These survivors were re-challenged 105 days later with $10^5$ EL4 cells subcutaneously (and also a group of C57BL/6 normal mice similarly treated), but all died, showing that no immunity had accrued from the first challenge with tumour cells.

In order not to confine experiments to the single lymphoma EL4, SB1 was tested in a similar situation to that previously described for EL4. C3H mice were immunized with SB1 live cells, Balb/c spleen cells or Balb/c thymus cells. Nine injections were given and the sera absorbed *in vivo* by giving 1 ml intraperitoneally to each of 20 Balb/c mice; these were bled after 4 h and 9 ml of absorbed serum recovered. This was tested for cytotoxicity on normal Balb/c lymphocytes and thymocytes and seen to be zero.

Groups of mice were challenged with $10^3$ SB1 cells 2 h after intraperitoneal injection of 0·5 ml of absorbed serum. It can be seen from Fig. 2 that the anti-thymus and anti-spleen sera were without any protective effect but serum against SB1 living cells gave a measurable delay of death from tumour growth. This and all subsequent tests indicated that it was much more difficult to protect against SB1 than EL4, but the relative difficulty and ease of protection provided two situations of value for tests to be described later. Using alloantisera against SB1, we have only with some difficulty been able to find a cytotoxic titre for SB1 target cells using sera absorbed to be

FIG. 2.—Protection of Balb/c mice against $10^3$ SB1 cells, by alloantibody made with SB1 live cells as immunogen in C3H mice, ●——●. Injections of saline, ○——○; of C3H anti-Balb/c spleen cell serum, △——△; of C3H anti-Balb/c thymus serum, □——□.

non-cytotoxic for normal Balb/c lymph node cells.

*Changes in test conditions*

Passive immunity for human patients would necessarily employ xenoantiserum, *in vitro* absorption and administration of the immunoglobulin fraction of serum. Before testing mouse tumour xenoantiserum, alloantiserum of known protective value against SB1 tumour cells was made and used in a series of tests leading to the following scheme.

Serum was obtained from 150 C3H mice which had received 8 injections of $10^7$ SB1 cells. The titre against Balb/c lymphocytes was 1/1200. Serum (62 ml) was absorbed overnight at 4°C at a level of 5 spleens/ml. This absorbed serum was unreactive with Balb/c lymphocytes (using guinea-pig complement and a 3 h incubation time). There was a cytotoxic titre against SB1 cells with this allo-derived serum using guinea-pig complement and a 3 h incubation period (see below). This is not shown because it was similar in magnitude to that already illustrated for EL4 (Davies and O'Neill, 1973). This absorbed alloantiserum was fractionated to yield 7·5 ml of immunoglobulin at 20 mg/ml. The results of a protection test using this material are shown in Fig. 3. The challenge dose was $10^3$ cells and the treatment consisted

FIG. 3.—Protection of Balb/c mice against SB1 cells, challenge dose $10^3$, i.p. Treatment, injections i.p. 4 h after challenge and again at 24 h intervals 3 times with: normal mouse serum (0·4 ml), ○——○; undiluted, absorbed alloantiserum from C3H mice (0·25 ml), △——△; Ig prepared from that antiserum, (2 mg) □——□; the same amplified by 0·2 mg chlorambucil, ●——●.

FIG. 4.—Protection of C57BL/6 mice against EL4 lymphoma cells, challenge dose $5 \times 10^4$. Treatment, injections i.p. 18 h after challenge and again at 24 h intervals 4 times with: normal rabbit serum (0·5 ml), ○——○; absorbed rabbit anti-EL4 serum (0·5 ml), △——△; Ig from the antiserum (5 mg), □——□; chlorambucil (75 μg), ●——●; chlorambucil (75 μg) followed by Ig from antiserum (5 mg), ▲——▲.

of 4 injections of serum, the first at 4 h and 3 times at 24 h intervals thereafter. It can be seen that fractionated antibody at 2 mg doses was somewhat less effective than whole antiserum (0·5 ml doses), but the effect of antibody could be magnified to make it more obvious by also injecting a small amount of chlorambucil (0·2 mg) an hour before antibody was injected, this being an insufficient quantity of drug to affect the issue when used alone. This topic is discussed more fully in a later report by Davies *et al.* (1974*a*).

### *The xenogeneic system*

An example of the final protection test system arrived at is as follows: A rabbit serum (R85/86) was used, resulting from 6 intravenous injections of $10^8$ living EL4 cells. The serum was heat inactivated (56°C for 30 min) and the yield of 38 ml was diluted 1 : 2 in saline, absorbed as indicated previously and fractionated to crude Ig with $AmSO_4$. The absorption data are given in Table I. Fig. 4 shows that protection can be obtained with xenoantiserum fractionated to Ig and absorbed *in vitro* to zero cytotoxicity for normal

TABLE I.—*Sequential Absorption of Rabbit Anti-EL4 Serum**

| | Absorbed with | Titre (original 1 : 1500) |
|---|---|---|
| 1 | 50 livers and spleens | 1 : 420 |
| 2 | 50 livers and spleens | 1 : 300 |
| 3 | 300 spleens | 1 : 200 |
| 4 | 720 spleens | 1 : 30 |
| 5 | 280 spleens | zero |

* Vol. of 136 ml at dilution 1 : 2, absorbed overnight at 4°C.

lymphoid cells. The time lapse between challenge and treatment can then be increased substantially over that which can be achieved with alloantiserum; in this example it was 18 h.

*Tumour specific antibody*

A series of protection tests showed that the potency of different antisera and the ease (or difficulty) with which they could be absorbed differed with the number of immunizing injections. This was tested as follows: rabbits (R96/98) were injected i.v. with $10^8$ live EL4 cells 5 times at 10 day intervals and bleedings of 40 ml taken before the second and each subsequent injection. The resulting 5 antisera were heat inactivated and cytotoxicity for normal cells was reduced to zero by absorption as shown in Table II. Using samples of these absorbed sera (checked with rabbit complement and a 3 h incubation time), the titres against EL4 cells varied as shown in Table II. These sera were then examined for their ability to give immunofluorescence of EL4 cells (checking for non-reactivity with normal C57BL/6 cells as control) using a goat Ig anti-rabbit Ig. The results showed no fluorescence after the first bleeding, a little after bleeding 2, maximal fluorescence after bleeding 3 and persisting submaximally in bleedings 4 and 5. Ammonium sulphate fractionation results are also shown in this Table.

The Ig from each bleeding was adjusted to 20 mg/ml for protection testing. A challenge dose of $5 \times 10^4$ EL4 cells was given intraperitoneally and 0·2 ml (4 mg) of antibody was given 4 times, the first being 6 h after challenge and then after 24, 48 and 72 h. Thus, the test was made less severe by reducing the time lapse from 18 h (as in the previous test) to 6 h, increasing sensitivity to seek smaller amounts of protective activity. It can be seen from Fig. 5 that the first bleeding had some activity but less than the second, the third was

TABLE II.—*Successive Bleedings from Rabbits Immunized with Mouse EL4 Lymphoma*

| Bleeding no. | No. of spleens/2 ml of 1 : 2 serum to absorb for zero titre against normal lymphocytes 1st | 2nd | 3rd | 4th | Residual titre* against EL4 cells | Yield (mg) of Ig |
|---|---|---|---|---|---|---|
| 1 | 10 | 10 | 0 | 0 | Nil | 113 |
| 2 | 10 | 10 | 10 | 0 | 1 : 12 | 133 |
| 3 | 10 | 10 | 10 | 5 | 1 : 30 | 105 |
| 4 | 10 | 10 | 10 | 10 | Nil | 104 |
| 5 | 10 | 10 | 10 | 0 | Nil | 122 |

* Incubation time 3 h using guinea-pig complement, cells not neuraminidase-treated.

maximal and the fourth and fifth had decreasing protective action.

The SB1 system gave a different result. Rabbits 93/95 were given 10 injections of $10^8$ SB1 cells intravenously and bleedings of 40 ml were taken 10 days before the second and each subsequent injection. Samples (1 ml) of these bleedings were diluted 1 : 2 in saline and the cytotoxicity absorbed out; the number of absorptions required to reduce cytotoxicity for normal cells to zero increased up to the third bleeding and subsequently decreased. With these data as a guide, bulk absorptions and fractionation to Ig were carried out. These Ig fractions were all adjusted to 20 mg/ml and used in a protection test where mice were challenged with $10^3$ cells intraperitoneally and given 4 mg of antibody (0·2 ml) 4 times in an " easy " system, *i.e.* treatment 2 h before, 4 h after and 28 and 56 h after challenge. The result is not illustrated; some protective activity could be detected using serum taken after the fifth and sixth injections only. Cytotoxicity against SB1 cells in sera having no residual reactivity against normal Balb/c lymphocytes was detectable only in the fifth bleeding.

*Class of antibody*

A pool of rabbit anti-EL4 sera (R140/145) was heat inactivated (56°C for

FIG. 5.—Protection of C57BL/6 mice against EL4 lymphoma, challenge dose 5 × $10^4$ cells. Treatment, injections i.p. 6 h after challenge and again 24, 48 and 72 h later, with 4 mg Ig from serum raised in rabbits. Rabbits bled 1 day before the second and each of 4 subsequent injections ($10^8$ EL4 cells each time i.v.). Controls (injected with buffered saline), ○——○; first bleeding, △——△; second, □——□; third, ●——●; fourth, ▲——▲; fifth, ■——■.

30 min), absorbed to provide only tumour specificity and a 12 ml sample passed through a Sephadex G–200 column (1 m × 5 cm, capacity 2·2 l) in 0·1 mol/l—$NH_4HCO_3$ at pH 8·0. Three pools were made from the 280 nm O.D. pattern—excluded (Pool A), retarded (Pool B) and included (Pool C), each reduced to the original vol (12 ml) and dialysed against saline; these we take to be IgM, IgG and albumin respectively in the gross sense.

These were assessed in a protection test where C57BL/6 mice were challenged with 5 × $10^4$ EL4 cells i.p. and treated with serum G–200 Sephadex fractions in 0·5 ml doses 18 h later and again on the following 3 days. The results of this test are not illustrated. Original serum (also 0·5 ml doses) provided for a 12 day increase in life span; the IgG fraction (Pool B) gave the same result. Pools A and C gave no protection against tumour growth; these mice died at 12 to 16 days after challenge, as did controls given normal mouse serum.

*Specificity*

Whereas we have assessed a number of mouse tumours for their usefulness in test systems of the kind described above, only EL4 and SB1 have been quoted in this paper. An experiment was carried out which showed lack of interaction between EL4 and SB1 systems; a further specificity control using the $H\text{-}2^b$ lymphoma ERLD which, like EL4, belongs to C57BL/6 mice (Old *et al.*, 1968) was also carried out. A rabbit anti-EL4 serum (R140/145) was used as described above to show that 0·5 ml given 4 times affected the growth of EL4 in C57BL/6 mice adversely and prolonged the lives of mice in this particular test for 9 days (prolongation from Day 15 to Day 24). In the same test, batches of mice were also challenged with ERLD (1 × $10^5$, i.p.); this dose and drug levels were based on ERLD background data of the kind shown in Fig. 1 for EL4 and SB1 and in the following paper describing the drug antibody (" DRAB ") effect (Davies *et al.*, 1974*a*). No protection against ERLD was obtained with anti-EL4 antiserum, and small doses of chlorambucil in addition did not show the amplification effect (as seen in Fig. 3 and 4), presumably because there was nothing to amplify.

### DISCUSSION

Protection tests as used by Gorer and Amos (1956) and many other workers in slightly different regimens and with different tumours have been described frequently in the literature. Thus, for example, fractions of EL4 cells were tested for the presence of tumour specific antigen; fractions were injected for active immunization of other mice to obtain allogeneic antisera to test for the presence of protective antibody (Davies, 1963). The results showed that tumour specificity (" X ") was located in or on the plasma membranes of EL4 tumour cells. Any clinical use of antibody, either alone or in combination with some other treatment, requires assessment in some animal model. There is no special novelty about the model described here, it was merely arranged, to suit our particular requirements, by extending (a) to another tumour (SB1), (b) from alloantisera to xenoantisera, (c) to the use of Ig rather than whole serum, and (d) to absorption *in vitro* instead of *in vivo*. These steps worked satisfactorily and have since been used with human material to provide tumour specific immunoglobulin (O'Neill, results to be published).

It will be seen that the number of injections to elicit the best anti-tumour response is both critical and difficult to determine. The number differs from one tumour to another, as seen by direct testing *in vivo*. It then becomes of great importance to have some *in vitro* method of assessment and it is interesting to see that both cytotoxicity for EL4 tumour cells (when it was zero for normal cells) and immunofluorescence ran parallel with protective activity when the three were tested on each of a series of antisera. A different number of injections required for the lymphoma SB1 also gave correlation between cytotoxicity and protection.

Gel filtration was used to show that the tumour specific xenoantibody responsible for protection was not IgM but was in the IgG fraction.

The adjustment of the tests from severe to relative ease as required was achieved by altering the challenge dose, the time lapse between challenge and treatment, or the number and size of treatment injections.

The amplification of the protective effect of antibody by drugs, which was touched on in this paper is expanded upon in a later communication (Davies *et al.*, 1974*a*).

We wish to thank Mr Richard Sugar for carrying out the immunofluorescence studies.

### REFERENCES

ALBERT, W. H. W. & DAVIES, D. A. L. (1973) H-2 Antigens on Nuclear Membranes. *Immunology*, **24**, 841.

AOKI, T., STÜCK, B., OLD, L. J., HÄMMERLING, U. & DEHARVEN, E. (1970) E Antigen: A Cell Surface Antigen of C57BL Leukemias. *Cancer Res.*, **30**, 244.

BOYSE, E. A., STOCKERT, E. & OLD, L. J. (1968) Isoantigens of the *H-2* and *Tla* loci of the Mouse. *J. exp. Med.*, **128**, 85.

DAVIES, D. A. L. (1963) Occurrence of X Antigenic Specificity in Histocompatibility Antigens prepared from Mouse Leukaemic Cells. *Br. J. exp. Path.*, **44**, 546.

DAVIES, D. A. L. (1966) Mouse Histocompatibility Isoantigens derived from Normal and from Tumour Cells. *Immunology*, **11**, 115.

DAVIES, D. A. L., BUCKHAM, S. & MANSTONE, A. J. (1974*a*) Protection of Mice against Syngeneic Lymphomata: 2, collaboration between Drugs and Antibodies. *Br. J. Cancer*, **30**, 305.

DAVIES, D. A. L., MANSTONE, A. J., BAUGH, V. S. G. & BUCKHAM, S. (1974*b*) The Tumour Specific Antigen of a Mouse Lymphoma. *Eur. J. Cancer*. In the press.

DAVIES, D. A. L. & O'NEILL, G. J. (1973) *In vivo* and *In vitro* Effects of Tumour Specific Antibodies with Chlorambucil. In *Immunology of Malignancy*. Ed. M. Moore, N. W. Nesbitt and Mary V. Haigh. *Br. J. Cancer*, **28**, Supp. I, 285.

GORER, D. A. & AMOS, D. B. (1956) Passive Immunity in Mice against C57BL Leukosis E.L.4 by Means of Iso-immune Serum. *Cancer Res.*, **16**, 338.

LECLERC, J. C., LEVY, J. P., VARET, B., OPPENHEIM, S. & SENIK, A. (1970) Antigenic Analysis of L Strain Cells: A New Murine Leukemia-associated Antigen, " L ". *Cancer Res.*, **30**, 2073.

OLD, L. J., STOCKERT, E., BOYSE, E. A. & KIM, J. H. (1968) Antigenic Modulation. *J. exp. Med.*, **127**, 523.

ROWLAND, G. F., EDWARDS, A. J., HURD, C. M. & SUMNER, M. R. (1971) Changes in Lymphoreticular Tissue During Growth of Adenocarcinoma. III. Plaque Forming Response in Lymph Node and Spleen. *J. natn. Cancer Inst.*, **47**, 321.

STAINES, N. A., O'NEILL, G. J., GUY, K. & DAVIES, D. A. L. (1973) Xenoantisera Against Lymphoid Cells: Specificity and Use in Monitoring Purification of Mouse and Human Histocompatibility Antigens. *Tissue Antigens*, **3**, 1.